# Chemical Composition and Potential Properties in Mental Illness (Anxiety, Depression and Insomnia) of Agarwood Essential Oil: A Review

**DOI:** 10.3390/molecules27144528

**Published:** 2022-07-15

**Authors:** Xiqin Chen, Canhong Wang, Qingqin He, Jian Feng, Deli Chen, Jianhe Wei, Yangyang Liu

**Affiliations:** 1 Key Laboratory of Bioactive Substances and Resources Utilization of Chinese Herbal Medicine, Ministry of Education & National Engineering Laboratory for Breeding of Endangered Medicinal Materials, Institute of Medicinal Plant Development, Chinese Academy of Medical Sciences and Peking Union Medical College, Beijing 100193, China; 18359033886@163.com (X.C.); Heqqin.77@gmail.com (Q.H.); wjianh@263.net (J.W.); 2 Hainan Provincial Key Laboratory of Resources Conservation and Development of Southern Medicine & Key Laboratory of State Administration of Traditional Chinese Medicine for Agarwood Sustainable Utilization, Hainan Branch of the Institute of Medicinal Plant Development, Chinese Academy of Medical Sciences and Peking Union Medical College, Haikou 570311, China; xinzhuangjianpo@163.com (C.W.); jianfenghn@126.com (J.F.); chendeli9999@163.com (D.C.)

**Keywords:** agarwood essential oil, insomnia, anxiety, mental illness

## Abstract

As a valuable medicinal herb and spice, agarwood is widely used in the fields of daily chemistry, traditional medicine, religion and literary collection. It mainly contains sesquiterpenes and 2-(2-phenylethyl)chromones, which are often used to soothe the body and mind, relieve anxiety, act as an antidepressant and treat insomnia and other mental disorders, presenting a good calming effect. This paper reviews the chemical composition of the essential oils of different sources of agarwood, as well as the progress of research on the sedative and tranquilizing pharmacological activity and mechanism of action of agarwood essential oil (AEO), and then analyzes the current problems of AEO research and its application prospects in the treatment of mental diseases.

## 1. Introduction

People used aromatic plants to prepare essential oils or fumigation baths thousands of years ago in ancient Egypt and ancient China to relax the body and prevent diseases [1]. Aromatic plant essential oil has pharmacological activities, such as anti-oxidation, bacteriostasis, calming, and nerve calming, according to modern pharmacology. It affects peoples’ mood, physiological state, and behavior by regulating the human autonomic nervous system, central nervous system (CNS), and (or) endocrine system via aromatic molecules [2,3,4]. Therefore, aromatherapy is frequently used as an adjunct therapy to treat chronic pain, depression, anxiety, insomnia, and other neurological diseases today [5,6,7].

Agarwood is resin-containing wood, formed in the *Aquilaria* or *Gyrinops* tree of the Thymelaeaceae family. The production of agarwood is unique in that healthy trees do not produce agarwood, but when they are damaged by the outside world, they activate their defense mechanism and produce a dark brown resin. These resin-laden woods are then called agarwood. Agarwood is rare and difficult to obtain, and the trees are often cut down indiscriminately, causing wild resources to be depleted. Currently, 21 species of the genus *Aquilaria* and nine species of the genus *Gyrinops* are listed in Appendix II of the Convention on International Trade in Endangered Species of Wild Fauna and Flora (CITES). Fortunately, with the widespread planting of *Aquilaria* spp. and the promotion of artificial-inducing methods, agarwood production has greatly improved.

Agarwood is a well-known aromatic traditional Chinese medicine that is listed in the Chinese Pharmacopoeia. At the same time, it is also a traditional precious medicinal material in Japan, India and other countries. Due to its distinct fragrance, agarwood is known as “the king of all incense, the first of all incense”, and it is used as incense in Buddhism, Hinduism, and Islam, as well as in other religious rituals. According to modern pharmacology, agarwood essential oil (AEO) has antioxidant, antibacterial, anti-inflammatory, air purifying, relaxing, and calming properties. Essential oils are frequently used in aromatherapy to treat anxiety, depression, insomnia, and other psychiatric illnesses [8,9,10]. However, the efficacy of AEO is related to its chemical composition. Essential oils obtained from different raw materials and different extraction methods have different chemical compositions. Hydro-distillation (HD), supercritical fluid carbon dioxide extraction (SFE), subcritical fluid extraction, cellulase-assisted extraction, microwave extraction method (MWE), and other methods are commonly used for extracting AEO [11]. Among them, HD and SFE methods are most commonly used.

The goal of this paper was to systematically review the progress of research on the chemical composition of AEO, as well as its sedative and tranquilizing pharmacological activity and mechanism of action, in order to analyze the current problems of AEO and its application prospects in the treatment of mental diseases. As of June 2022, the information reported in this study derived from scientific databases, such as Web of Science, PubMed, CNKI. The search keyword was “agarwood”, and the articles were manually filtered according to needs.

## 2. The Chemical Composition of AEO

AEO contains numerous chemical components, primarily sesquiterpenoids, (2-phenylethyl)chromones, aromatics, fatty acids, etc. [12].

### 2.1. Sesquiterpenoids

Sesquiterpenoids are natural terpenoids containing 15 carbon atoms in a molecule composed of three isoprene units. There are 210 sesquiterpene compounds, including those of the agarofuran-type, agaro-spirane-type, guaiane-type, and eudesmane-type, which have been isolated and identified as of this writing by researchers, both domestically and internationally [13,14,15,16]. According to modern pharmacology, sesquiterpene components of agarwood have good biological activity in the central nervous system, respiratory system, digestive system, and other systems [17].

### 2.2. (2-Phenylethyl)chromones

2-(2-phenylethyl)chromones are synthesized by substituting the phenylethyl fragment at the C-2 position of the chromogenic ketone [18]. One of agarwood’s primary active components, chromone, has been isolated and found to have 240 different subunits. It has anti-inflammatory and anti-tumor properties, is neuroprotective, and has acetylcholinesterase, tyrosinase, and glucosidase inhibitory actions, among other properties [19,20,21,22]. It is worth noting that there are no 2-(2-phenylethyl)chromones in the essential oil of incense extracted by hydro-distillation. However, the extracted AEOs obtained by supercritical fluid extraction and microwave-assisted extraction methods usually contain Flidersiachromone, 6-methoxy-2-(2-phenylethyl)chromone, 4H-1-Benzopyran-4-one chromone, and a few other semi-volatile 2-(2-phenylethyl)chromones.

### 2.3. Low-Weight Aromatic Compound

Low-weight aromatic compounds are also important components of AEO, and they are frequently regarded as the primary source of aroma in AEO. It has been reported that more aromatics can be detected in heated incense or AEO. Takamatsu discovered that heated cleavage of 2-(2-phenylethyl)chromones in agarwood produces low molecular weight aromatic compounds [23].

### 2.4. Fatty Acids

Fatty acids are less commonly reported in AEO. Chen discovered that essential oil extracted from healthy white wood contained up to 59% n-hexadecanoic acid and oleic acid, whereas when the healthy tree was subjected to injury to form agarwood, its fatty acid composition decreased and sesquiterpenes and 2-(2-phenylethyl)chromones increased. It can be seen that the lower the fatty acid content of AEO, the higher the quality of the oil [24].

## 3. Factors Affecting the Chemical Composition of AEO

As can be seen from the previous section, the main components of AEO are sesquiterpenes, 2-(2-phenylethyl)chromones, aromatics, and fatty acids. However, the chemical composition of AEO can be influenced by different sources of agarwood (including species, origin, age, agarwood-induction method, agarwood-induction time), and AEO extraction methods. Table 1 shows the results of chemical composition analysis of various AEOs at home and abroad in recent years.

### 3.1. Plant Species

At present, it is reported that 22 species of plants in the genus *Aquilaria* and seven species in the genus *Gyrinops* can produce agarwood, and the main species of commercially available agarwood are *A. malaccensis*, *A. sinensis*, *A. crassna*, etc. From Table 1, it can be seen that both *A. malaccensis* and *A. crassna* essential oils have higher content of fatty acid compounds than *A. sinensis* essential oil obtained by water distillation. At the same time, among all the AEOs extracted by hydro-distillation, only the essential oil of “Chi-Nan” (an *A. sinensis* variant) contains a small amount of 2-(2-phenylethyl)chromones [31]. It can be seen that the chemical composition of the AEO extracted from different *Aquilaria* trees is different. However, at present, the chemical compositions of the essential oils of different species have not been subjected to comprehensive and systematic comparison, making it difficult to distinguish the quality of their advantages and disadvantages.

### 3.2. Agarwood-Induction Methods

The three categories of artificial induction of agarwood are physical ways, chemical methods, and biological methods. Wild agarwood is created when an agarwood tree gets harmed by outside forces, such as wind, rain, lightning, insects, and so forth. The physical method is to induce agarwood after the tree has been subjected to external physical injuries, such as cutting, chiseling, trunk breaking, nailing, and fire branding. The chemical method is to induce agarwood with plant hormones, NaCl, H_2_O_2_, and various solutions. The biological method involves inducing agarwood by inoculating with microorganisms. Chen compared the chemical composition of AEOs obtained by different methods and discovered that 2-(2-phenylethyl)chromones were twice as abundant in AEO formed by the fungi-inoculation method as they were in AEO formed by the burning-chisel-drilling (BCD) method [32]. Liu used ten different chemical inducers and three traditional agarwood-inducing methods, namely, the partly-trunk-pruning method (PTP), the BCD method, and the fungi-inoculation method (FI), to induce 7-year-old *A. sinensis* trees to produce agarwood. He finally found that the essential oil extraction rate of agarwood induced by the whole-tree agarwood-inducing technique (Agar-Wit) for 6 months was much higher than that induced by the other three methods over more than 12 months, and that the AEO induced by the Agar-Wit contained more aromatic components [25]. In conclusion, different agarwood-induction methods have a significant impact on the extraction rate and chemical composition of AEO, and Agar-Wit, which is a competitive inducing technique, can reduce the time taken in inducing the production of agarwood, while effectively improving agarwood quality.

### 3.3. Extraction Method

From Table 1, it can be seen that the composition of AEO compounds obtained from different extraction methods varies greatly. The extraction rate of AEO by hydro-distillation (HD) is low, typically 0.1–0.5%, but the relative content of sesquiterpenes is higher and the relative content of small-molecule aromatics is lower, which could be due to AEO volatilization loss caused by continuous high temperature during the HD process. In contrast, due to the mild conditions, the extraction rate in SFE is high, up to 0.8–5%, with more aromatic components, and, due to the strong solubility of supercritical fluid, many chromones can also be extracted. It is worth noting that the composition of the AEOs extracted by different solvents varies; even for the HD method, 95% ethanol can present more aromatic components and a large number of chromones. Meanwhile, Geng observed that the microwave-assisted extraction method produced the greatest variety of compounds in incense essential oil among different extraction methods using the same solvent [30]. As can be seen, in practical production applications, a suitable extraction method should be selected based, on the type of AEO required.

## 4. The Use of AEO to Treat Mental Illness

According to ancient Chinese literature, agarwood has the traditional effect of “removing bad energy and making people awake”, and is mostly used to improve sleep through burning and heating. At the same time as AEO is aromatherapy’s most commonly used essential oil for its calming and tranquilizing traditional efficacy in aromatherapy, it has also shown good auxiliary treatment of mental illness, mainly regarding anxiety, depression, and insomnia.

### 4.1. Anti-Anxiety Effects

There are many causes of anxiety, and it is generally believed that anxiety disorders are due to disorders in the CNS that regulate the anxiety process, such as a decrease in GABA and monoaminergic neurotransmitters, and similar, resulting in dysfunction of the neurotransmitter system [33]. Benzodiazepines, selective serotonin reuptake inhibitors (SSRIs) and serotonin-norepinephrine reuptake inhibitors (SNRIs) are clinically recommended as first-line pharmacological treatments for anxiety disorders [34,35]. The anxiolytic effects of benzodiazepines are mediated by promoting the binding of GABA to GABA_A_ receptors after binding to the corresponding receptors, resulting in a decrease in neuroexcitability and alleviation of anxiety symptoms. SSRIs and SNRIs, for example, exert anxiolytic effects by inhibiting the reuptake of 5-HT and norepinephrine. In the treatment of patients with long-term anxiety, the problem of side effects of medication has to be considered. Therefore, people prefer long-term effective treatments, such as psychological counseling, relaxation therapy, acupuncture therapy, and aromatherapy, just to mention a few [36].

Chronic restraint stress (CRS) is a commonly used method to induce psychological and physiological stress responses, which can cause a range of behavioral and physiological changes in the nervous and endocrine systems, and studies have demonstrated that stressful situations can induce the production of anxiety [37]. Wang examined the anxiety state of animals by restraining stress-induced mice using a series of behavioral methods such as the elevated plus maze (EPM) test, the light dark exploration (LDE) test, and the open field (OF) test [10]. The experimental results showed that the AEO administration group was able to dose-dependently improve the anxiety symptoms of mice, and significantly increased the time, distance, and entry of mice with open arms in the EPM experiment. The LDE experiment increased the residence time of mice in the light chamber. The effect of AEO at 40 mg/kg was comparable to that of the positive drug diazepam (2.5 mg/kg), which proved the significant anxiolytic effect of essential oil [10]. Japanese scholars found that both heated AEO and monomeric components were effective in reducing motor activity and acting as sedatives in mice at a range of doses through vapor inhalation and administration of benzylacetone, calarene, and α-gurjunene compounds, the main aromatic analogues in AEO alone. They further verified that different benzylacetone-like compounds also have sedative activity, the strength of which depends on the functional group on the carbon chain, the substituent on the benzene ring, and different combinations of functional groups and substituents [8,38]. At the same time, the benzene extract of agarwood had a decreasing effect on spontaneous movements in mice and was able to significantly prolong pentobarbital sodium-induced sleep time. These results suggest that the benzene extract of agarwood has a strong CNS inhibitory effect, while the compounds jinkoh-eremol and agarospirol, isolated from the benzene extract, have a tranquilizing effect similar to chlorpromazine, in addition to inhibiting CNS activity [39].

Of interest is that Buagafuran, a new class 1.1 anti-generalized anxiety disorder drug obtained by structural modification of an agarofuran-type compound isolated from agarwood, has completed its phase IIa clinical trial [40]. It is evident that AEO and several monomeric compounds isolated from agarwood have significant anxiolytic activity.

### 4.2. Anti-Depressant Effects

The pathogenesis of depression is related to many factors, and low function of the monoaminergic nerves (5-OH, NA) in the brain may be one of the causes of depression. The main antidepressants used in clinical practice today are monoamine reuptake inhibitors, monoamine oxidase inhibitors, and other antidepressants, but data shows that about half of depressed patients have no effect after the first dose, and subsequent treatment is not promising [41]. Therefore, it remains important to find new and effective antidepressants.

By performing tail suspension (TS) test and forced swimming (FS) test on stressed mice, Wang showed that a certain dose of AEO (20 and 40 mg/kg) had similar efficacy to paroxetine (10 mg/kg) in TS and FS experiments and significantly reduced the immobility time of mice; thus, surmising that AEO has good antidepressant efficacy [10]. Wang also discovered that the Twenty Flavored Agarwood Pill was able to shorten the immobility time of mice in TS and FS experiments and alleviate the depressive symptoms of mice when they observed the behavioral changes of the pill on desperate mice [42].

Huang grouped 40 depressed patients into two groups: the control group was treated with basic therapy (psychological guidance and antidepressant medication), and the treatment group was treated with agarwood fumigation therapy. The agarwood fumigation treatment was on the basis of lighting an incense wire twice a day in both groups for 2 weeks as a course of treatment, for a total of two courses of treatment. The results showed that the treatment group’s total effective rate was 85% and the control group’s total effective rate was 55%. It showed that aromatherapy was effective in improving the clinical symptoms of depressed patients and reducing the CES-D (Depression Scale) and HAMD (Hamilton Depression Scale) scores, with good clinical effects [43].

### 4.3. Sleep-Promoting Effects

Sleep is an active process that occurs mainly in the CNS and the associated neurotransmitters and cytokines. Based on the various factors involved in this physiological process, the causes of insomnia are extremely complex. There are two main theories about the pathogenesis of insomnia: (1) dysfunction of the hypothalamic-pituitary-adrenal cortex system (HPA) and dysregulation of the neuroendocrine system, due to stress and other factors; (2) dysregulation of the central neurotransmitters associated with sleep-wake effects. DA and NE are neurotransmitters that promote and maintain wakefulness, while inhibitory neurotransmitters, such as 5-HT and GABA, have sedative-hypnotic functions [44,45]. Sedative-hypnotic drugs are a class of drugs that have a depressant effect on the CNS and can be divided into benzodiazepines, barbiturates, and other classes of drugs. according to their chemical structure. However, in addition to the side effects of drugs for insomnia, long-term medication can have problems, such as drug resistance and addiction. In contrast, agarwood is a traditional sedative and tranquilizer with almost no drug resistance [9].

Wang used different extracts (aqueous extracts, alcoholic extracts, and volatile oils) of the agarwood to synergize the suprathreshold and subthreshold hypnosis experiments with sodium pentobarbital. It was found that the agarwood extract and AEO significantly increased the sleep rate and prolonged sleep time in mice compared to the blank group, and the AEO also significantly shortened the sleep latency in mice, suggesting that agarwood extract and AEO have strong sedative-hypnotic effects [46]. Hou found that oral and nasal inhalation administration of agarwood tablets to mice in small (0.4 g for 1 mouse), medium (1.2 g for 1 mouse), and large (3.6 g for 1 mouse) dose groups significantly shortened sleep latency and prolonged sleep time of the suprathreshold dose of sodium pentobarbital, and that the medium dose group significantly increased the number of animals sleeping with the subthreshold dose of sodium pentobarbital [47]. Liang used normal mice and insomnia mice induced by PCPA (300 mg/kg) and investigated the effects of agarwood incense inhalation on sleep and mood by various behavioral assays [48]. The results showed that the inhalation of agarwood gas (0.3 g for 6 mice and 0.5 g for 6 mice) significantly promoted the sleep of normal mice and significantly improved the quality of life and motor ability of insomniac mice. Meanwhile, it has been shown that after one to two weeks of diazepam and AEO administration to insomniac mice, the hypnotic effect of diazepam administration decreased, or even disappeared completely, in the diazepam-administered group, while there was no significant drug resistance in the agarwood-administered group [9].

By treating 120 insomnia patients in groups, with the experimental group receiving fumigation with agarwood each night before bedtime and the control group receiving fumigation with propanol that did not affect sleep, Lei found that fumigation treatment with agarwood significantly improved the sleep quality and reduced the severity of insomnia in patients with insomnia disorder, as well as being able to improve mood disorders, such as anxiety and depression [49]. The above studies have shown that AEO has a significant sedative-hypnotic effect with few side effects and no significant drug resistance, making it an effective alternative therapy for insomnia.

**Figure 1:**
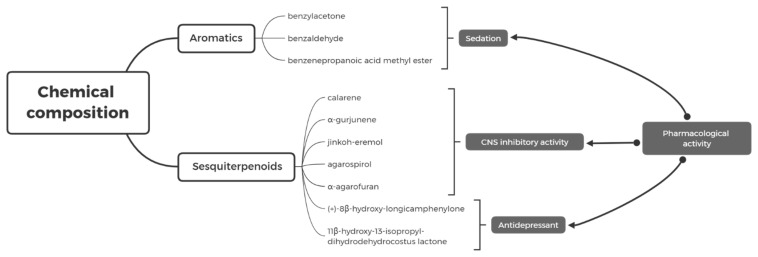
Pharmacological activities of the main monomer components in Agarwood.

In summary, AEO has significant sedative and tranquilizing effects (anxiolytic, antidepressant, and pro-sleep) (Table 2), while several of its monomeric compounds, such as benzylacetone, calarene, and jinkoh-eremol, have significant central nervous system activity, which may be the material basis for the sedative and tranquilizing effects of AEO (Figure 1) and have the potential to be developed into new psychotropic drugs.

## 5. Mechanism of Action of AEO in the Treatment of Mental Illness

Monoaminergic neurological functions projecting to the prefrontal cortex are enhanced early in stress, whereas chronic long-term stress depletes monoamine neurotransmitters and reduces neuronal functions [50]. As a result of stress-induced increases in cortisol, brain-derived neurotrophic factor (BDNF) expression is reduced in the brain, leading to neuronal cell death (especially in the hippocampus), deterioration in brain function and an increased incidence of psychiatric disorders, such as depression, anxiety, and insomnia. With age, the loss of DA and reduced expression of BDNF can lead to the development of central neurodegenerative diseases, such as Parkinson’s disease (PD) and Alzheimer’s disease (AD) [51]. Based on the physiological basis of psychiatric disorders, potential mechanisms of action of AEO for the treatment of psychiatric disorders include modulation of the central nervous system/endocrine system and related neurotransmitters, antioxidant effects, acetylcholinesterase inhibitory activity, neurocytoprotection, anti-inflammatory and analgesic effects.

### 5.1. Inhibition of HPA Axis Hyperactivity

Under prolonged or continuous chronic stress, the HPA axis is regulated through a series of neuroendocrine regulations. Neurons in the paraventricular nucleus (PVN) of the hypothalamus produce and release corticotropin-releasing factor (CRF), which promotes the secretion of adrenocorticotropic hormone (ACTH) in the anterior pituitary gland. Repeated stress eventually leads to the secretion of more cortisol, and excessive levels of corticosterone can cause many psychiatric disorders, such as anxiety and depression [44]. There is substantial evidence that elevated CRH and an overactive HPA axis contribute to depression and anxiety symptoms in humans [52], and, therefore, the HPA axis modulatory effect is a major mechanism in the treatment of psychiatric disorders.

The CUMS depression model caused hyperexcitability of the HPA axis and increased the expression of ACTH, corticosterone, and CRH genes in rats, whereas inhaling the essential oil of agarwood-elecampane lowered serum levels of ACTH and corticosterone, restored levels of 5-OH, DA, NE, and ACHE in the hippocampus and regulated the hyperexcitability of the HPA axis [53]. The anxiolytic and antidepressant effects of AEO were further investigated in a stress-induced mouse model by Wang [10]. Stress induced elevated blood levels of inflammatory factors and nitric oxide (NO), which, in turn, led to hyperactivity of the HPA axis [10]. AEO dose-dependently suppressed serum levels of cytokines, such as interleukin IL-1α, IL-1β, and IL-6. It significantly reduced neuronal nitric oxide synthase (nNOS) mRNA levels in the cerebral cortex and hippocampus, inhibited nNOS protein levels in the hippocampus, decreased gene expression of the gene regulating CRF upstream of the HPA axis, and decreased ACTH and corticosterone (CORT) concentrations downstream of the HPA axis in a dose-dependent manner [10]. This suggests that the antidepressant and anxiolytic effects of AEO are mainly mediated by reducing the hypersensitivity of the HPA axis.

### 5.2. Associated Neurotransmitter Changes

Changes in the levels of neurotransmitters and their metabolites in the brain are thought to be closely associated with the onset of psychiatric disorders. GABA is an inhibitory neurotransmitter that mediates synaptic transmission in the central nervous system. By binding to its receptors, it increases the permeability of cell membranes to potassium and chloride ions, particularly the massive inward flow of chloride ions, and the occurrence of postsynaptic membrane hyperpolarization, inhibiting the occurrence of action potentials and exerting postsynaptic inhibition [54,55]. Inadequate GABA in the brain can keep neurons in a state of impulsive excitation for an extended period of time, which is one of the major causes of physiological insomnia. However, GABA itself cannot pass the blood-brain barrier (BBB), and GABA in the brain is produced by Glu in the brain as raw material, so the ratio of Glu to GABA is often used in experiments as an indicator to objectively evaluate the excitation and inhibition status of central nervous system functions. Of the monoamine neurotransmitters, 5-HT, DA, and NE, 5-HT is thought to be associated with the etiology of many disease states, such as insomnia, depression, anxiety, and panic disorder; and DA and NE with the maintenance of the waking state [56,57].

AEO has certain efficacy on PACA-induced insomnia rats. By measuring the content of neurotransmitters, it was found that the 5-OH content in the brain of rats in the model group was significantly reduced, while AEO administered to the group could significantly increase 5-HT content, reduce DA and NE content, significantly increase GABA content and reduce Glu content, resulting in a significant decrease in Glu/GABA [44]. Wang found that AEO not only promoted the synthesis and release of GABA, and increased the expression of GABAA receptors, but also promoted the inward flow of chloride ions, which may be one of the mechanisms of sedative-hypnotic effects of incense in clinical practice [9,58]. At the same time, the use of the GABA receptor benzodiazepine site competitive antagonist flumazenil resulted in flumazenil effectively blocking the sleep-promoting effect of AEO. The latter effect increased with increase of the dose, which proved that the sedative and sleep-promoting effect of the essential oil was mainly through the regulation of the GBBAergic system [9].

Kao found that serum serotonin (5-OH) levels in mice increased from 551 ± 344 ng/mL to 952 ± 334 ng/mL after 7 days of Kynam (also known as Chi-Nan) administration to mice, and the data suggested that Kynam may show sedative and antidepressant effects, due to elevated serum serotonin levels [59]. Meanwhile, microarray analysis of RNA profiles in mouse brains revealed that five mood-related pathways, including dopaminergic synapses, 5-hydroxytryptaminergic synapses, GABAergic synapses, long-term depression, and neuroactive ligand-receptor interactions, were affected by Kynam [59]. These experimental results suggest that the anxiolytic and antidepressant effects of agarwood may be associated with increased 5-OH levels and multiple neuroactive pathways in mice.

### 5.3. Neuroprotective Effect

Wang used H_2_O_2_ to oxidatively damage hippocampal nerve cells, and the experimental results showed that high, medium, and low doses of AEO applied to oxidatively damaged neurons all increased hippocampal nerve cell survival rates to varying degrees, indicating that AEO has neuronal protective activity [58]. He created an in vitro model of antidepressant-induced P12 cell injury using CORT and discovered that several compounds isolated from agarwood had significant protective effects on P12-injured cells [60]. Yang found that seven chromones isolated from agarwood expressed significant neuroprotective activity and greatly improved cell survival in glutamate-induced and corticosterone-induced P12 chromophobe cells and human U251 glioma cells, while the compound 6,8-dihydroxy-2-[2-(30-hydroxy-40-methoxyphenyl)ethyl]chromone exhibited neuroprotective activity comparable to that of the positive drug fluoxetine, with an 82.2% and 86.9% increase in cell viability for both types of cells, respectively [61]. Brain-derived neurotrophic factor (BDNF) is essential for neuronal survival, development, and synaptic plasticity. The Japanese scholar Jun-ya Ueda found that 70% ethanolic extract of agarwood and a single sesquiterpene compound (4R,5R,7R)-1(10)-spirovetiven-11-ol-2-one, isolated from agarwood, both showed significant induction of BDNF mRNA expression regarding central nervous activity. It is clear that BDNF’s neuroprotective effects are primarily manifested by increased survival of damaged nerve cells and the expression of BDNF [62]. It is evident that agarwood manifests its neuroprotective effects mainly by enhancing the survival of damaged nerve cells and the expression of BDNF.

### 5.4. Antioxidant Effect

The oxidative stress hypothesis is one of the many hypotheses for central degenerative disease that have been accepted by most scholars. When exposed to oxidative stress, the body produces a large amount of free radicals, which damage proteins, lipids, DNA, and other macromolecules, resulting in a decrease in enzyme activity, metabolic disorders, and apoptosis [63]. As a result, antioxidant therapy is extensively used in psychiatric disorders such as PD, AD, and anti-depression and anxiety disorders [64,65].

The treatment of PC12 cells with H_2_O_2_ by Xiong showed a significant decrease in cell viability, impaired intracellular antioxidant enzyme activity and a significant accumulation of reactive oxygen species, while treatment with AEO reduced intracellular reactive oxygen species levels and significantly enhanced intracellular antioxidant enzyme activity, indicating that the AEO significantly reduced oxidative damage in PC12 cells [63]. Lee found that agarwood alcohol extract reduced oxidative damage in the hippocampus of mice subjected to repeated stress, demonstrating the neuroprotective effect of agarwood alcohol extract on oxidative damage in the hippocampus [64]. The significant antioxidant effects of agarwood alcoholic extracts can also be seen in a mouse model of isoproterenol-induced myocardial infarction, indicating that agarwood alcoholic extracts can significantly scavenge free radicals, inhibit oxidative stress, and exert anti-apoptotic effects [66].

### 5.5. Anti-Inflammatory Effect

Inflammation is the underlying cause of many diseases, and there is growing evidence that neuroinflammatory changes, including microglia, play an important role in the pathogenesis of psychiatric disorders, with microglia hyperactivation and neuroinflammation being one of the main causes of neuronal cell death [67]. Epidemiological investigations have demonstrated a 50% reduction in the risk of AD in patients on long-term non-steroidal anti-inflammatory drugs (NSAIDs) [68], Wessel found that participants with high levels of inflammatory markers had high levels of anxiety (accompanying depressive states) through a nine-year-long evaluation of 2867 patients with depression and anxiety [69], and clinical trials have shown that anti-inflammatory drugs can be used as both monotherapy for depression [70]. Therefore, inflammation plays an important role in the development of psychiatric disorders.

Agarwood has excellent anti-inflammatory activity in vitro and in vivo. According to Li, an ethanolic extract of agarwood had a significant inhibitory effect on xylene-induced auricular swelling in mice and could effectively improve the increase in capillary permeability caused by acetic acid in mice [71]. Jin-Seok Lee found that pretreatment of BV2 microglia with the dichloromethane fraction of agarwood significantly reduced lipopolysaccharide-induced overproduction of NO, COX-2, PGE2 and interleukin 1β and alleviated microglia-derived neuroinflammatory responses [72]. Furthermore, many sesquiterpenes and chromones isolated from incense showed anti-inflammatory activity and inhibited lipopolysaccharide-induced NO production in RAW 264.7 cells [20,73]. It is clear that incense and its constituents have potent anti-inflammatory properties.

### 5.6. Acetylcholinesterase Inhibitory Activity

Acetylcholine is a neurotransmitter in the body that is rapidly hydrolyzed in tissues by cholinesterase to form acetate and choline, which can block nerve signals. In 1792 Janowsky proposed the hypothesis of hypercholinergic-adrenergic dysfunction as the pathogenesis of depression, and recent studies have shown that various cholinesterase inhibitors have antidepressant-like properties in the forced swimming test in mice [74,75]. Furthermore, acetylcholine deficiency is a major cause of AD, and acetylcholinesterase inhibitors improve cognitive function in patients with cognitive impairment. As a result, inhibiting acetylcholinesterase activity is regarded as an effective therapeutic approach for the treatment of depression and AD [76,77].

Many of the monomeric components of agarwood, such as sesquiterpenes and chromones, have good acetylcholinesterase inhibitory activity. The sesquiterpene compound (4S,5R,7R)-11,12-dihydroxy-eremophila-1(10)-ene-2-oxo-11-methyl ester was isolated from the ethyl acetate extract of agarwood with a strong inhibitory activity of 42.9 ± 0.6% [78]. Shao also isolated several sesquiterpene compounds in agarwood at a concentration of 50 μg/mL as 33.3–54.2% inhibition of acetylcholinesterase. Yang used the Ellman method to test the acetylcholinesterase inhibitory activity of 34 compounds isolated from “Chi-Nan” and found that most of the sesquiterpenes and chromones had some acetylcholinesterase inhibitory activity, especially the sesquiterpenes of the eremophane type, with inhibition rates ranging from 30.5% to 61.9% at a concentration of 50 μg/mL [79]. Yang found that among the monomeric compounds isolated from *A. crassna*, three epoxy-2-(2-phenylethyl)chromones had significant acetylcholinesterase inhibitory activity [80]. Wei Li counted at least 69 compounds isolated from agarwood with varying degrees of acetylcholinesterase inhibitory activity [21]. It is clear that agarwood contains a large number of natural acetylcholinesterase inhibitors with therapeutic potential for neurodegenerative diseases.

### 5.7. Other Pharmacological Actions

Essential oils have pharmacological effects that are mediated not only by the skin and respiratory system, but also by the sense of smell. According to research, aroma can signal the olfactory system and influence the limbic system of the brain through smell, stimulating the brain to release neurotransmitters. Odors can cross the BBB faster and more effectively than the oral medications currently used to treat psychiatric disorders, resulting in a coordinated and rapid response [51]. As a result, many odors have the potential to be new treatments or may contribute to the current treatment of psychiatric disorders. As an aromatic medicine, agarwood has many aromatic small-molecule components, which may be one of the mechanisms of AEO in the treatment of psychiatric disorders. Metabolomic data suggests that abnormalities in biomarker metabolism occurred between the control and agarwood inhalation groups, suggesting that agarwood intervention may mediate insomnia and mood disorders through the tryptophan metabolic pathway [81]. In addition, a total of 27 compounds of AEO were found to have potential targets through target prediction, BBB and plasma protein binding analysis by network pharmacology, including neuromodulation-related pathways, such as the neuroactive ligand-receptor interaction pathway, cAMP signaling pathway, 5-hydroxytryptaminergic pathway, cholinergic pathway, and dopaminergic pathway. The Therapeutic Target Database (TTD) was used to collect therapeutic targets for sleep disorders, anxiety, and depression and to map them with the predicted targets of compounds. It was found that 11 compounds had potential effects on 30 therapeutic targets for sleep disorders, anxiety, and depression [82].

In summary, AEO is simple to administer, produces little drug resistance, and has good pharmacological effects on anxiety, insomnia, and depression. It also has neuroprotective, antioxidant, and anti-inflammatory effects, modulates the HPA axis and related neurotransmitters, and has potential applications for central degenerative diseases, such as AD, PD and other psychiatric disorders, as predicted by network pharmacology and other aid.

## 6. Discussion

The chemical components of agarwood have been researched domestically and internationally since the 1950s. Agarwoods have so far yielded 240 chromones, 210 sesquiterpenes, and a few additional compounds that have been isolated and named. However, the majority of papers on AEO are on the analysis and comparison of different AEO chemical components; there are few reports on the separation and identification of AEO chemical components. The chemical composition of AEO consists primarily of sesquiterpenoids, 2-(2-phenylethyl)chromones, small molecules of aromatic substances, and fatty acids, and the source of agarwood (including species, origin, age, agarwood-induction method, agarwood-induction time) and essential oil extraction methods can affect the chemical composition of AEO. The AEOs’ chemical makeup can be used to determine legitimacy and processes used in extraction. AEOs derived via HD and SFE, for example, differ significantly. Additionally, chemical composition can be used to separate AEOs from various sources. AEOs produced from “Chi Nan” and common agarwood differ from one another. There is currently no clear specification grade of AEO. We primarily sort out existing research on the chemical composition of AEO, with the hope of making some modest contributions to the future classification of AEO grade. The disadvantage is that current research on the essential oils of various types of agarwood is primarily based on a comparison of chemical composition, and a comparative study of pharmacological activity is still insufficient to explain the best of the agarwood, “Chi-Nan”, “Hainan agarwood”, “Yellow wax agarwood”, and its scientific connotation as a valuable spice.

As a valuable spice, in addition to its unique, elegant, and unpredictable fragrance, agarwood has the traditional effect of calming and tranquilizing the mind, and nowadays AEO remains one of the essential oils commonly used in aromatherapy to soothe the body and mind, and is applied to relieve anxiety and depression, and to treat insomnia and other mental disorders. According to modern pharmacology, AEO may act as a sedative and tranquilizer (anti-anxiety, anti-depressant, and promoting sleep) by inhibiting HPA axis overreaction, suppressing nNOS protein levels in the hippocampus, reducing gene expression in the cerebral cortex and hippocampal CRF, lowering serum levels of ACTH and CORT (as evidenced in mice), and modulating neurotransmitters, such as 5-OH, DA, NE, and so on. At the same time, AEO has good neuroprotective, anti-inflammatory, and antioxidant properties. The network’s pharmacological results also suggest that it could be used to treat central degenerative diseases, such as AD, PD, and other psychiatric disorders.

However, the application of AEO at home and abroad has not been widely developed. At present, in addition to aromatherapy essential oil products on the market, most AEO is used as raw materials for perfume and fragrance water, and its clinical application is almost non-existent. Its unique pharmacological advantages for the treatment of mental illnesshave not yet been tapped into, which may be related to the fact that early AEO is expensive and research into its pharmacological efficacy and mechanism of action has not been deep enough. Fortunately, due to the artificial cultivation of *A. sinensis* and artificial agarwood-induction technology breakthroughs, with a large number of agarwood raw materials entering the market, the price of AEO has fallen. Therefore, research into the application of AEO in treating psychiatric disease and its in-depth development should be the current focus.

## Figures and Tables

**Table 1:** Composition of chemical constituents in different AEOs.

Agarwood Species	Origin	Agarwood-Induction Methods	Extraction Method	Chemical Composition	Ref.
*A. sinensis*	China	Agar-Wit (6 month)	HD	Aromatics: 6.44~10.07% Sesquiterpenes: 47.43~52.78% Fatty acids: 0.50~0.98%	[25]
*A. sinensis*	China	FI (12 month)	HD	Aromatics: 2.70% Sesquiterpenes: 34.84% Fatty acids: 0.16%	[25]
*A. sinensis*	China	BCD (20 month)	HD	Aromatics: 2.10% Sesquiterpenes: 57.58% Fatty acids: 0.36%	[25]
*A. sinensis*	China	PTP (28 month)	HD	Aromatics: 1.51% Sesquiterpenes: 52.03% Fatty acids: 0.69%	[25]
*A. sinensis*	China	Wild (unkown)	HD	Aromatics: 2.34~2.39% Sesquiterpenes: 62.35~71.48% Fatty acids: 0.06~0.35%	[25]
*A. sinensis*	China	BCD (unkown)	HD	Aromatics: 10.63% Sesquiterpenes: 68.68%	[26]
*A. sinensis*	China	BCD (unkown)	SFE	Aromatics: 12.46% Sesquiterpenes: 23.78% chromones: 29.42%	[26]
*A. sinensis*	China	unkown	SFE	Aromatics: 0.22~1.15% Sesquiterpenes: 8.93~26.10% chromones: 23.95~53.27% Fatty acids: 0~0.38%	[27]
*A. sinensis*	China	unkown	HD	Sesquiterpenes: 54.97~57.53% Fatty acids: 1.79~2.42%	[27]
*A. malaccensis*	Malaysia	unkown	HD	Aromatics: 2.81~6.26% Sesquiterpenes: 37.66~75.43% chromones: 0~0.38% Fatty acids: 0~16.376%	[27]
*A. crassna*	Vietnam	FI (3 year)	HD	Aromatics: 2.54~12.47% Sesquiterpenes: 61.06~72.37% Fatty acids: 6.57~8.46%	[28]
*A. crassna*	Thailand	hammering nails into its trunk	HD	Aromatics: 1.79% Sesquiterpenes: 92.86% Fatty acids: 2.84%	[29]
*A. crassna*	Thailand	hammering nails into its trunk	SFE	Aromatics: 0.8% Sesquiterpenes: 34.52% Fatty acids: 5.91%	[29]
*A. sinensis*	China	insect infestation	HD (95% ethanol)	Aromatics: 5.12% Sesquiterpenes: 12.34% chromones: 31.62% Fatty acids: 23.83%	[30]
*A. sinensis*	China	insect infestation	SE (95% ethanol)	Aromatics: 11.6% Sesquiterpenes: 7.72% chromones: 21.68% Fatty acids: 19.72%	[30]
*A. sinensis*	China	insect infestation	SFE (95% ethanol)	Aromatics: 9.64% Sesquiterpenes: 13.58% chromones: 34.48% Fatty acids: 28%	[30]
*A. sinensis*	China	insect infestation	MAE (95% ethanol)	Aromatics: 11.02% Sesquiterpenes: 21.6% chromones: 30.81% Fatty acids: 25.77%	[30]
*A. sinensis* (Chi-Nan)	China	hole-drilling (18 month)	HD	Aromatics: 0.98% Sesquiterpenes: 90.28% chromones: 0.93%	[31]

**Table 2:** Summary of the sedative and tranquilizing pharmacological activity experiments of agarwood.

Pharmacological Activity	Extracts/Compounds (Administration Route)	Function/Mechanism	Ref.
anti-anxiety (restraint stress-induced anxiety)	AEO: 10, 20 and 40 mg/kg Positive drug: diazepam 2.5 mg/kg i.p.	In EPM, LDE and OF test, the AEO (40 mg/kg) dose was equivalent to the positive drug diazepam (2.5 mg/kg). Mechanism: inhibition of HPA axis hyperactivity.	[10]
anti-depressant (restraint stress-induced depression)	AEO: 10, 20 and 40 mg/kg Positive drug: paroxetine 10 mg/kg i.p.	40 mg/kg AEO showed the best efficiency among the tested dosages with a comparable effect to that of paroxetine (10 mg/kg).	
sedative effect	AEO: 400 μL Optimal dose: benzylacetone 0.1%, calarene 0.17%, α-gurjunene 1.5% inh	On mice’s spontaneous motor activity, both AEO and monomers had dose-dependent benefits.	[8]
sedative effect	Benzylacetone: 4 × 10^−4^, 4 × 10^−3^, 4 × 10^−2^, 4 × 10^−1^, or 4 mg. inh	Significantly reduced autonomic motor activity (4 × 10^−4^ mg had the strongest sedative effect).	[38]
CNS inhibitory activity	agarwood benzene extract: 400 mg/kg Positive drug: trazepam 10 mg/kg i.p.	It could reduce spontaneous exercise and increase sleep time induced by hexabarbituric acid, and the intensity of its effect (400 mg/kg) was greater than that of trazepam (10 mg/kg).	[39]
anti-depressant	Chenxiang pellets: 1.0 g/kg Positive drug: Fluoxetine 0.01 g/kg i.g.	Chenxiang pellets could significantly reduce the immobility time of mice during tail suspension and forced swimming. Mechanism: It could be related to increasing 5-HT content in the mouse hippocampus, inhibiting the expression of s1c6a4 and HTR1A mRNA in the mouse hippocampus, and improving the function and excitability of the central 5-HT system.	[42]
anti-depressant	agarwood incense (twice a day, 1 month) inh	Agarwood aromatherapy had a positive clinical effect on CES-D and HAMD scores in patients with depression.	[43]
sedative-hypnotic effects	AEO: 50 μL and 100μL (80 °C, 30 min) inh	AEO could significantly increase the sleep rate, sleep time, and sleep latency in mice. Mechanism: It actedas a sedative and hypnotic by modulating GABA receptor gene expression and improving GABA receptor function.	[9,46]
anti-anxiety promoting sleep	Chenxiang pellets: 0.4 g, 1.2 g, 3.6 g (150 °C 20 min) inh	① In EPM, LDE, and OF tests, AEO demonstrated significant anti-anxiety activity. ② Three doses of pentobarbital sodium could significantly reduce sleep latency while increasing sleep time.	[47]
promoting sleep (induced by PCPA)	agarwood incense: 0.3 g for 6 mice and 0.5 g for 6 mice inh	It could improve the insomnia of mice. Mechanism: it was mainly related to the regulatory effect of aloe gas inhalation on Glu/GABA.	[48]
promoting sleep	Agarwood: 0.5 g, 3 h inh	Agarwood treatment could significantly improve the quality of sleep and the severity of insomnia in patients with insomnia, as well as anxiety, depression, and other emotional disorders.	[49]

Note: i.p.: intraperitoneal injection; i.g.: intragastrical administration; inh: inhalation administration.

## Abbreviations

AEO	Agarwood essential oil
CNS	central nervous system
HD	Hydro-distillation
SFE	supercritical fluid carbon dioxide extraction
MWE	microwave extraction method
PTP	partly-trunk-prunning method
BCD	burning-chisel-drilling method
FI	fungi-inoculation method
Agar-Wit	whole-tree agarwood-inducing technique
SE	Soxhlet extraction
MAE	Microwave-assisted extraction
SSRIs	selective serotonin reuptake inhibitors
SNRIs	serotonin-norepinephrine reuptake inhibitors
CRS	chronic restraint stress
EPM	the elevated plus maze test
LDE	the light dark exploration test
OF	the open field test
TS	tail suspension test
FS	forced swimming test
HPA	hypothalamic-pituitary-adrenal cortex system
BDNF	brain-derived neurotrophic factor
PD	Parkinson disease
AD	Alzheimer disease
ACTH	adrenocorticotropic hormone
CRF	corticotropin-releasing factor
nNOS	neuronal nitric oxide synthase
BBB	the blood-brain barrier
Glu	Glutamic Acid
